# Screening for suicide risk in medical settings: From research to implementation

**DOI:** 10.1192/j.eurpsy.2021.473

**Published:** 2021-08-13

**Authors:** L. Horowitz

**Affiliations:** Office Of The Clinical Director, National Institute of Mental Health, Bethesda, United States of America

**Keywords:** medical settings, Suicide, Screening, suicide risk

## Abstract

**Introduction:**

Suicide is an international public health problem and a leading cause of death for youth and adults, worldwide. Prevention efforts in health care systems create opportunities for identifying medical patients with occult suicidality. Detecting suicide risk among patients in medical settings can be a challenge, but successful suicide risk screening programs have been demonstrated in hospital settings.

**Objectives:**

This presentation will discuss how a suicide risk screening tool that was developed for the pediatric emergency department was tested and then implemented in other medical settings in order to leverage healthcare providers as partners in combating the public health crisis of youth suicide..

**Methods:**

Implementation and quality improvement projects in various medical settings that have adapted the ASQ will be described. Effective management of pediatric patients that screen positive for suicide risk and how mental health clinicians can best be utilized in efficient ways will also be discussed.

**Results:**

Average time to administer the ASQ was 20 seconds. Positive screen rates across ED, inpatient and outpatient settings ranging from 2-14% equating to one additional psychiatric consultation per week. The ASQ Toolkit was developed to help medical providers implement screening including scripts for nurses, flyers for parents and a brief suicide safety assessment (ASQ BSSA) to operationalize next steps for patients at risk.

**Conclusions:**

The medical setting is a key venue for youth suicide risk detection and linking patients with effective interventions. Mental health clinicians have a role in guiding non-mental health providers in the identification and management of patients found to be at risk.

**Disclosure:**

No significant relationships.

